# Chardonnay Marc as a New Model for Upcycled Co-products in the Food Industry: Concentration of Diverse Natural Products Chemistry for Consumer Health and Sensory Benefits

**DOI:** 10.1021/acs.jafc.2c04519

**Published:** 2022-11-21

**Authors:** Roberta R. Holt, Daniela Barile, Selina C. Wang, John P. Munafo, Torey Arvik, Xueqi Li, Fanny Lee, Carl L. Keen, Ilias Tagkopoulos, Harold H. Schmitz

**Affiliations:** † Department of Nutrition, University of California, Davis, Davis, California 95616, United States; ‡ Department of Food Science and Technology, University of California, Davis, Davis, California 95616, United States; § Department of Food Science, University of Tennessee, Knoxville, Tennessee 37996, United States; ∥ Sonomaceuticals, LLC, Santa Rosa, California 95403, United States; ∇ PIPA, LLC, Davis, California 95616, United States; ⊥ Department of Computer Science and Genome Center, USDA/NSF AI Institute for Next Generation Food Systems (AIFS), University of California, Davis, Davis, California 95616 United States; ◇ March Capital US, LLC, Davis, California 95616, United States; ○ T.O.P., LLC, Davis, California 95616, United States; # Graduate School of Management, University of California, Davis, Davis, California 95616, United States

**Keywords:** oligosaccharides, upcycle, phenolics, flavanols, flavor, computational modeling, cardiometabolic health, microbiota, Chardonnay, byproducts

## Abstract

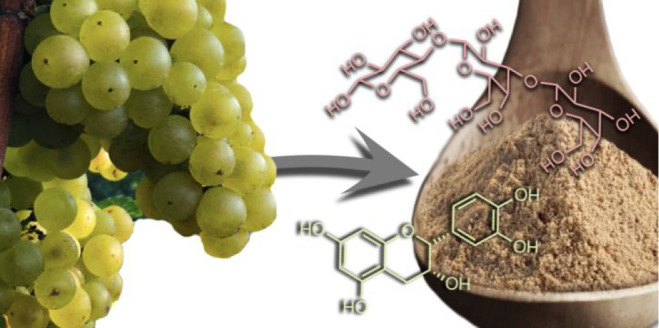

Research continues to provide compelling insights into potential health benefits associated with diets rich in plant-based natural products (PBNPs). Coupled with evidence from dietary intervention trials, dietary recommendations increasingly include higher intakes of PBNPs. In addition to health benefits, PBNPs can drive flavor and sensory perceptions in foods and beverages. Chardonnay marc (pomace) is a byproduct of winemaking obtained after fruit pressing that has not undergone fermentation. Recent research has revealed that PBNP diversity within Chardonnay marc has potential relevance to human health and desirable sensory attributes in food and beverage products. This review explores the potential of Chardonnay marc as a valuable new PBNP ingredient in the food system by combining health, sensory, and environmental sustainability benefits that serves as a model for development of future ingredients within a sustainable circular bioeconomy. This includes a discussion on the potential role of computational methods, including artificial intelligence (AI), in accelerating research and development required to discover and commercialize this new source of PBNPs.

## Introduction

The past few decades have seen advances in science and technology enabling the systematic study of plant-based natural products (PBNPs) and their potential bioactivity in humans. This includes greater understanding of PBNPs not classified as essential nutrients, yet having considerable potential for impact on human health. Research on PBNPs encompassing broad classifications, such as the flavonoids, phenolic acids, and carotenoids, has yielded extensive insights into potential health benefits associated with diets rich in certain plant-based ingredients. In this context, epidemiological research has raised awareness of these potential benefits, while also adding phrases such as “French Paradox” and “Mediterranean Diet” to the lexicon of public health. Coupled with learnings from dietary intervention trials, the totality of this data has led to dietary recommendations that include dietary patterns with higher intakes of PBNPs.^1,2^ Dietary patterns such as the Mediterranean diet and Dietary Approaches to Stop Hypertension (DASH) include high intakes of plant-based foods providing a diverse array of PBNPs, including mono- and polyunsaturated fatty acids, essential vitamins and minerals, amino acids, fiber, and phenolics.^2,3^ Building on this foundation, recent data from the landmark COcoa Supplement and Multivitamin Outcomes Study (COSMOS) trial demonstrated that the daily intake of a specific class of PBNPs, in this case monomeric and polymeric flavanols from cocoa, by older individuals reduces risk for cardiovascular morbidity and mortality.^4^ COSMOS demonstrates the potential for precision nutrition strategies to improve public health via dietary intake of specified PBNPs. To realize the full potential for public health benefits from such an approach, there is a need for data on the health impacts of specific PBNP compounds and classes throughout the lifespan. Importantly, for these nutrition approaches to have maximum impact, a more comprehensive understanding of food composition as it pertains to PBNPs in terms of both content and strategies for the preservation of bioactivity during processing and manufacturing of food will help aid in the identification of new product and ingredient opportunities.

In addition to health, PBNPs often drive flavor and sensory perceptions in foods and beverages.^5,6^ The commercial relevance of these sensory attributes (e.g., taste, smell, and texture) has driven advances regarding the isolation and characterization of PBNPs, while also adding enormously to the general library of known compounds occurring in nature.^7^ In contrast, relatively high concentrations of PBNPs can lend potentially negative sensory attributes with severe product application limitations.^8^ Attributes that include astringency and bitterness may prevent broader implementation of these PBNP ingredients toward health and food applications. This is a potential limitation given their agricultural origin and the opportunity to upcycle byproducts from these materials into nutrient dense ingredients. There is increasing interest to reduce food waste and loss on a global basis for the achievement of long-term environmental sustainability, as well as food and health security.^9^ Therefore, food waste streams represent an opportunity to reincorporate PBNPs back into the food system.^7^

Chardonnay marc (or pomace) is currently an underutilized byproduct of Chardonnay winemaking, consisting of seeds, stems, and skins obtained after pressing of fruit that has not undergone fermentation. The 2021 California wine crush alone generated 121,600 tons of Chardonnay wine grape marc derived from more than 3.8 million tons of wine grapes on a fresh weight basis at an average price of $975 per ton.^10^ As a byproduct of wine production, there is potential interest for Chardonnay marc as a co-product or a desirable secondary product, similar to that of whey as a co-product for cheese making, particularly given the economic impact of such a waste stream to the producer. Recent research into the natural products chemistry of Chardonnay marc has revealed additional diversity of bioactive compounds potentially relevant for human health, as well as desirable sensory attributes appropriate for the development of new foods and beverages. Given the scale of this industrial sector, converting this underutilized Chardonnay marc byproduct into a valuable ingredient for use in food and beverage products represents an extraordinary opportunity for creating a sustainable circular bioeconomy within the winemaking sector. In this perspective, we examine recent advances in the characterization of natural products in Chardonnay marc, along with the potential of Chardonnay marc PBNPs to improve human health, as well as sensory characteristics of food and beverage products. We highlight the opportunity this combination of attributes enables in terms of scaling its use, with a corresponding contribution to environmental sustainability. In addition, we discuss an early application of predictive analytics of Chardonnay marc PBNPs, and the role data science can play in the acceleration of research and development of PBNP ingredients into a healthier and more sustainable food system.

## Chardonnay Marc: An Underutilized Source of PBNPs

Chardonnay originates from the famed Burgundy region of France during the Middle Ages near a village of the same name (Latin for “a place of thistles”) in Maĉon.^11^ Recent genotyping places its parentage from Pinot noir and Gouais blanc (also known as Heunisch Weiss, Rebula Stara, and Belina Starohrvatska) with additional analyses suggesting a shared parentage between these two varietals.^12−15^ Historically considered a poor variety, planted by peasants, efforts were made to ban Gouais blanc.^16^ What attributes Gouais blanc passed on to its well-known prodigy are currently unknown. In the United States, California is the largest producer of wine, with Chardonnay as its top-planted grape. Chardonnay was present in California by the late 1880s, but as the end of prohibition in 1933, had a limited presence in the state. Viticulturist Dr. Harold P. Olmo from the University of California, Davis, and USDA ARS plant pathologist Dr. Austin Goheen are credited with the development of virus-tested clones that produced higher yields in a variety of California’s climate zones. Their work allowed for an increase in Chardonnay acreage from 986 acres in 1968 to over 7000 acres by the mid-1970s; noteworthy as by 1976 a 1973 vintage of Chateau Montelena, a California produced Chardonnay, from Napa Valley, outperformed some of France’s best in a blind test tasting in Paris.^11^ Chardonnay is predominately grown in the cooler coastal regions and is the largest in tonnage of wine grapes crushed in California, representing 16.0% of the wine grapes crushed in 2021 at approximately 619,000 tons.^10^

The winemaker dictates the harvesting of wine grapes as they pursue the optimization of desired sensory attributes. Production of white wine involves pressing the must after crushing of the fruit in order to separate the juice for fermentation into wine. The remaining solids, known as marc or pomace, contain seeds, stems, pulp, and skins. Depending on the varietal, marc may account for 10–20% (w/w) of the grapes, estimated as 1 kg of marc generated for every 6 L of wine produced.^17^ Chardonnay marc is a notably underutilized byproduct in this industry. The production scale of this material has driven considerable interest in upcycling of the marc, including applications relevant to agriculture, bioenergy, and extraction of industrial value added products such as enzymes and biopolymers.^18^

In general, new environmental policies and strategies are being developed and implemented to drive more sustainable byproduct management in the food and agriculture sector; however, the wine industry still relies heavily on traditional composting and livestock feed, thus yielding limited value creation to the producer in terms of financial returns. New approaches to upcycling of wine grape marc could therefore enable value creation opportunities for the wine industry based on a circular bioeconomy. This opportunity requires innovative research initiatives that focus on further elucidation of grape marc composition, along with the potential of PBNPs to form the basis for new applications at an industrial scale. In the context of potential health applications via inclusion in foods and beverages, previous research has demonstrated that wine grape PBNPs from red and white varietal marcs, to include Chardonnay, possess antioxidant and antimicrobial properties.^19,20^ These properties offer a glimpse into the potential for wine grape marcs to serve as value-added ingredients in foods and beverages.

Given the scale of Chardonnay production in California and globally, Chardonnay marc could represent an opportunity for significant value creation as a food and beverage ingredient. Traditional uses of marc include distillation into spirits and as animal feed; with marc feed fortification shown to improve the antioxidant capacity and fatty acid profile of meat.^21−23^ Additional applications of marc into the human diet can include the use of flours and seed products, such as oils and extracts. Marc can provide fiber, natural sugars, minerals, protein, lipids, and (poly)phenols.^24^ However, differences in grape varietals, agricultural, postharvest handling, and processing practices can affect the composition of PBNPs for many food products.^25^ Therefore, it is likely that varietal, extent of ripening, fermentation, and subsequent processing for food/dietary application can all impact PBNP composition. For example, Corte-Real et al. suggested that stabilizing the water content to reduce microbial growth is important as microbial spoilage of grape marc significantly reduces the phenolic content, while also highlighting marc from white wine grape varietals as being richer in polyphenols compared to red wine grape counterparts.^26^ Finally, food-safe processing of grape marc requires specialized processing equipment and regulatory registrations to comply with global food safety initiative (GFSI) requirements.

## Phenolic Compounds

**Figure 1:**
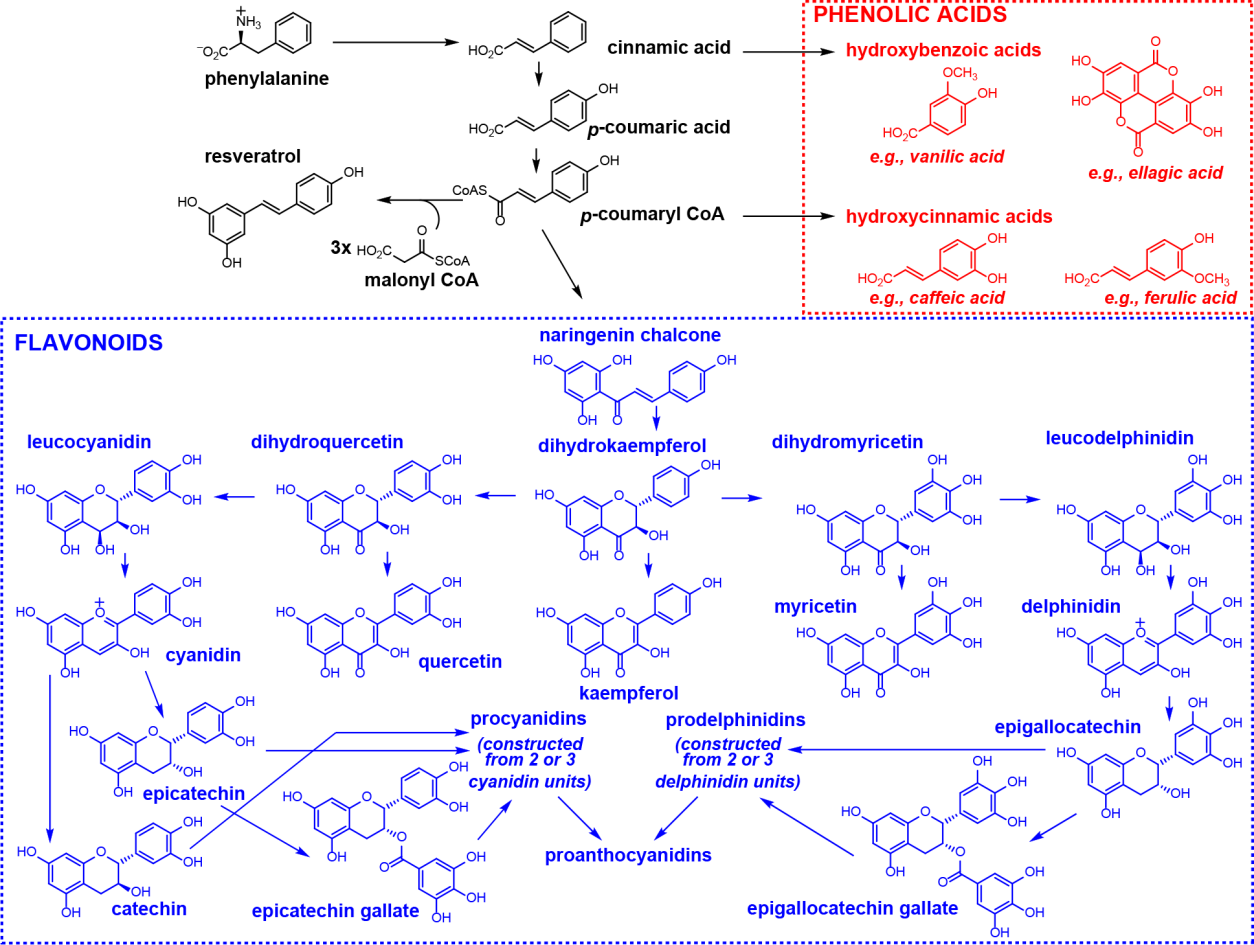
Production of Chardonnay grape berry phenolic acids and flavonoids from the metabolism of phenylalanine in the phenylpropanoid pathway.^171,172^

Wine grape phenolics and polyphenolics originate from phenylpropanoid pathway deamination of the aromatic amino acids phenylalanine and tyrosine to cinnamic acid.^27,28^ In grape marc, this includes the phenolic acids, hydroxycinnamic and benzoic acids, polyphenols such as flavonoids (e.g., flavanols and flavonols), stilbenes, tannins, and lignin^29,30^ (Figure 1). These PBNPs are diverse in structure and bioactivity potential, with substantial variation among wine grapes as well as other plant foods. Moreover, while any specific plant food can include a number of flavonoid subclasses, often only one will predominate. For example, cocoa flavanols are a significant source of (−)-epicatechin (epicatechin), (+)-catechin (catechin), and procyanidins up to ten degrees of polymerization (DP). In addition to epicatechin and catechin, tea is a significant source of gallocatechins, their gallic acid esters, and the polymers theaflavins, thearubigins, and theasinensins.^31,32^ Differences in processing of flavanol-rich foods, such as marc, can also affect bioactivity. For example, prolonged heat treatment epimerizes epicatechin to less bioactive (−)-catechin. Importantly, specialized HPLC methodologies, such as chiral chromatography, are needed to detect this epimerization within foods, which have reduced bioactivity, as epicatechin cannot be resolved from its epimer using traditional methods.^33−35^ This is an important consideration in terms of precision nutrition approaches, given that current recommendations for healthy dietary patterns are broad and diverse in terms of plant foods, and therefore the specific flavonoids present in overall diets would vary accordingly.

Chardonnay marc contains hydroxybenzoic and -cinnamic acids, flavonols and flavanols, including oligomers of the monomeric flavanols, epicatechin, catechin, and other derivatives of catechin known as the procyanidins and proanthocyanidins. The so-called “French Paradox” of a low prevalence of coronary heart disease in the French population with a high fat diet, albeit controversial, was first attributed to the intake of all wine,^36^ and later to the dietary intake of flavonoids present in red wine.^37^ Unlike red varietals, Chardonnay marc is not a significant source of anthocyanins, with lower flavonol levels; however, it can provide similar, or even greater, amounts of flavanols.^38,39^ For comparison, Rodriguez Montealegre et al. reported total monomeric flavanols in skin for Spanish Chardonnay, Cabernet Sauvignon, and Merlot at 28 ± 65 mg/kg, 23 ± 86 mg/kg, and 38 ± 21 mg/kg, respectively and in seeds at 730 ± 207 mg/kg, 430 ± 85 mg/kg, and 520 ± 128 mg/kg, respectively. Moreover, procyanidin B2 (i.e., two units of epicatechin) levels in Chardonnay and Cabernet Sauvignon seeds were 33 ± 5.8 mg/kg, and 41 ± 4.4 mg/kg, respectively. Also in Chardonnay seeds, a 2-fold greater amount of procyanidin B1 (i.e., one unit each of catechin and epicatechin) than Cabernet Sauvignon seeds (380 ± 5.8 mg/kg versus 150 ± 54 mg/kg, respectively).^40^ A consistent finding is that greater amounts of (poly) phenols are present in the seeds compared to the skins for all wine varietals^26,41−43^ (Table 1).

**Table 1:** Range of Phenolic and Polyphenol Content of Chardonnay Marc Skin and Seeds

phenolic	skin^a^	seeds^a^	ref
Hydroxybenzoic Acids			
ellagic acid	0.02	1.4–140	(167, 173)
gallic acid	0.04–12	0.5–152	(39, 43, 164, 166−168, 173)
gentistic acid	0.6		(167)
*p*-hydroxybenzoic acid	0.5–1.4	0.8	(164, 167)
vanillic acid	0.03–1.5	0.26–15.5	(43, 168)
caffeic acid	0.04–0.06	0.09	(164, 167)
caftaric acid	0.09–7.6	2.0	(38, 165, 166)
chlorogenic acid	0.06–0.2	0.3	(164, 167)
*p*-coumaric acid	0.07–1.8		(164, 167)
coutaric acid	0.8–7.6		(38, 165)
fertaric acid	0.1		(38)
ferulic acid	0.2–1.4		(164, 167)
syringic acid	0.07		(164)
dihydroquercetin-3-*O*-glucoside	1.5–1.6		(164)
isorhamnetin-3-*O*-glucoside	0.05–2.2		(38, 164)
Hydroxycinnamic Acids			
kaempferol	0.06		(167)
kaempferol-3-*O*-galactoside	3.6–7.7		(164, 165)
kaempferol-3-*O*-glucoside	0.8–26.8		(38, 164, 165)
kaempferol-3-*O*-glucuronide	3.1–5.0		(164, 165)
myricetin		0.2	(167)
myricetin-3-*O*-glucoside	1.7–1.8		(164)
myricetin-3-*O*-glucuronide	1.5–1 0.7		(164)
quercetin	0.6–1.6	1.5	(164, 167)
quercetin-3-*O*-galactoside	14.6		(165)
quercetin-3-*O*-glucoside	1.7–68.6	1.3	(38, 165, 166)
quercetin-3-*O*-glucuronide	2.5–17.8	0.2	(38, 165, 166)
quercetin-3-rutinoside (rutin)	1.2–6.2	0.2	(165−167)
quercetin-3-xyloside	2.2		(165)
Flavanones			
naringin	0.08	0.2	(167)
hesperetin	0.05	0.08	(167)
Flavones			
apigenin		0.06	(167)
Flavanols			
(+)-catechin	0.04–60.0	3.3–1247	(38, 39, 43, 164−168)
(−)-catechin gallate	0.08	8.3–12.2	(43, 167)
(−)-epicatechin	0.01–44	5.1–1940	(38, 39, 43, 164−168)
(−)-epicatechin-3-*O*-gallate	0.2–0.3	3.9–62.6	(38, 43, 164, 166)
(−)-epigallocatechin	0.4–38.5	42.6	(43, 164)
(−)-epigallocatechin gallate	0.25–2.2	0.47–5.6	(43, 167)
(−)-gallocatechin	94		(43)
(−)-gallocatechin gallate	1.2	9.9–26.6	(43, 167)
Proanthocyanidins			
dimer B1	48.9	38	(38, 165)
dimer B2	37.0	3.3–251	(165, 166)
dimer B3		5.2	(38)
dimer B4		7.1	(38)
galloylated dimers		980	(173)
total dimers (not galloylated)		89–5540	(173, 174)
trimer	18.5	10.3–22800	(165, 173, 174)
galloylated trimers		840	(173)
tetramers		10–1460	(173, 174)
galloylated tetramers		560	(173)
pentamer		81.5–1170	(173, 174)
hexamer		62.3–640	(173, 174)
heptamer		65–650	(173, 174)
octamer		61–420	(173, 174)
nonamer		52–230	(173, 174)
decamer		69–320	(173, 174)

a In mg/100 g.

## Fiber and the Gut Microbiota

PBNPs present in wine grape marc also include a complex carbohydrate fraction that can significantly impact both the health properties and sensory attributes of the material. Dietary fiber is a collective term for nonstarch and nondigestible polysaccharides, which includes the noncarbohydrate lignin, composed of phenolic compounds covalently bound to polysaccharides.^43−45^ The general class of PBNP dietary fiber is complex with its definition constantly debated and refined, primarily due to the constraints and limitations in analytical chemistry, which impede a proper characterization and quantification of its individual components.^45^ In an effort to harmonize the nomenclature, the Institute of Medicine (IOM) defined *dietary fiber* as nondigestible carbohydrate and lignin that is intrinsic and intact in plants, *functional fiber* as isolated nondigestible carbohydrate that has a functional physiological effect in humans, and lastly, *total fiber* as the sum of dietary and functional fiber.^46^ Yet, an important aspect of fiber, not captured by these definitions, is the effect of fiber on the human gut microbiota. For instance, some fibers undergo extensive fermentation by the resident gut bacteria producing short chain fatty acids (SCFAs), whereas other fibers simply provide viscosity aiding to thicken the intestinal contents and slow transit time.^46^ Short chain fatty acids such as acetate, propionate, and butyrate are produced in the colon, where they are used as preferential energy sources by intestinal cells. They also be systemically taken up via the portal vein to exert effects in organs and tissues as signaling molecules.^47^

States of fiber deficiency have not been established, with adequate intakes based on the median level of intake that lowers cardiovascular risk, which for adults is set at 14 g per 1000 kcal.^46^ Yet, it is known that diets poor in fiber lead to reduced diversity in microbial populations and shift the overall metabolism from saccharolytic to proteolytic patterns, with the appearance of undesirable metabolites originating from extensive colonic microbial amino acid fermentation (known as putrefaction).^48^ Additionally, detrimental functional changes have been demonstrated to take place in the intestine such as increased degradation of the protective mucin layer by the microbiota.^49^ Therefore, the intestinal microbiota, made up of trillions of microbial cells, is increasingly being recognized as an important determinant of health, with nondigestible carbohydrates representing the prototypical ingredient that can provide a health benefit to the consumer over and above the inherent nutritional content.^50^ It is not surprising that the gastrointestinal tract and its resident microbiota have become an important area of study for the development of novel PBNP. Commensal bacteria can provide a physical barrier that displaces pathogens from receptor binding sites on epithelial surfaces, can compete for available nutrients, and may also secrete antimicrobial agents that protect the host against exogenous pathogens.^51^ To more accurately identify important carbohydrates for the gut, the Sonnenburg Lab (Stanford, CA) proposed the term “Microbiota-Accessible Carbohydrates” or MACs.^52^ This nomenclature, if widely adopted by the scientific community, will allow us to differentiate the functional roles of indigestible carbohydrates and will prompt further investigation on the level of metabolic activity that a specific food can be expected to produce within a given microbiota, increasing our understanding of the “prebiotic selectivity” concept.^55^

Grape skin flour and seeds are rich sources of dietary fiber that ranges from 24 to 60%, with seeds containing greater amounts of fiber compared to the skin.^53^ In terms of prebiotic activity, structure dictates the selectivity since consumption of a specific carbohydrate depends on the presence of genes for the breakdown into monosaccharide building blocks and their metabolism by gut bacteria. Overall, a higher diversity of monosaccharides is desirable as it is associated with greater selectivity in utilization by commensal bacteria. Cellulose, hemicellulose, pectin, and lignin make up the cell wall components of grape skin and seeds. In terms of structural properties, cellulose is a linear homopolysaccharide made of β(1–4) repeating glucose units, whereas hemicellulose is a heteropolysaccharide containing different monosaccharides in the backbone (both hexose and pentose sugars) further decorated by β(1–2), β(1–3), and β(1–6) linked side chains of galactose, arabinose, and glucuronic acid. Finally, pectin has an α(1–4)-linked galacturonic acid backbone substituted in certain regions with α(1→2) rhamnose units with additional side chains made of a diversity of monosaccharides (galactose, mannose, glucose, rhamnose, and xylose).^54^ A recent analysis of seedless Chardonnay marc reports a 30% greater amount of cellulose compared to seeds isolated from marc (86.0 mg/g dw versus 60.6 mg/g dw, respectively) with 3-fold higher levels of hemicellulose in the seed component versus the seed-free marc fraction (78.6 mg/g dw versus 26.1 mg/g dw, respectively). Lignin contributes to the seed’s woody structure and was 7-fold greater in the seed component compared to marc without seed (364 mg/g dw versus 52 mg/d dw, respectively).^43^ While pectin characterization was not part of that study, a recent optimization of pectin extraction from Chardonnay marc produced a polysaccharide fraction containing 55.7% homogalacturonan and 35.2% rhamnogalacturonan I (RG-I) with short chains or single units of arabinose and galactose (RG-1).^55^

**Figure 2:**
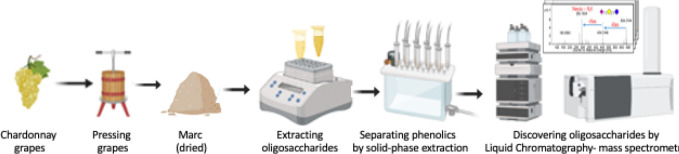
Quantification can be conducted using peak heights or areas (label-free quantification) or by isotopic labeling strategies. Through analysis of tandem-MS data and comparison with authentic and high-purity analytical standards, specific oligosaccharide structures present in a sample can be deduced.

Unlike other dietary components, the impact of dietary fiber on health is not dependent on its absorption and distribution to the tissues, but rather its impact on the alimentary tract, interaction with other dietary components, and the gut microbiota. Ultimately, the magnitude of its effect depends on the specific polysaccharide composition. For example, poorly fermented fibers, such as cellulose, provide bulk and have a laxative effect, whereas viscous fibers have a more profound influence in the upper gastrointestinal (GI) tract by slowing the transit of food. This reduces glucose absorption and inhibits bile acid reabsorption to lower plasma cholesterol levels.^45,56^ Moreover, gut microbial metabolites, such as SCFAs produced from fermentable fibers can positively regulate cardiometabolic health.^57^ While randomized, double-blind clinical trials are increasingly validating the health benefits derived from prebiotic use, the molecular mechanisms by which these approaches exert such an effect remain, for the most part, obscure. Oligosaccharides are one class of carbohydrates that has been rapidly gaining attention thanks to their multifunctional health properties. Oligosaccharides have a low degree of polymerization (e.g., 3 to 15 monosaccharide units), yet are a diverse class of compounds fermentable by select species of the gut microbiome. They are naturally present in mammalian milks and in many plants, after in vivo synthesis or through the hydrolysis of higher polysaccharides, glycoproteins, and glycolipids. Recent interest from the nutritional community in oligosaccharides derives from their unique added health benefits. For example, human milk oligosaccharides (HMOs) are known to play a critical role in the growth, stimulation, and cognitive development of the infant and, perhaps most remarkably, the establishment of the intestinal microbiome.^58^ Several HMOs also share common structural motifs with oligosaccharides on the infant’s intestinal epithelia known to be receptors for pathogens. The presence of such structures imply that HMOs provide a defensive strategy, acting as decoys to prevent binding of pathogens to epithelial cells, thereby protecting infants from disease.^59^ Comprehensive studies characterizing HMOs support the hypothesis that their structural complexity is the basis for a multitude of biological functions, the full range of which is only now beginning to be unraveled. In particular, bacterial fermentation of oligosaccharides requires specific activities of enzymes belonging to the glycosyl hydrolase family 20 (e.g., β-d-galactosidase, α-l-fucosidase, *N*-acetyl-β-d-hexosaminidases, etc.).^60^ Remarkably, only a few select *Bifidobacterium* strains, such as *B. longum* subsp. longum, *B. infantis*, *B. breve*, and *B. bifidum*, possess the genetic capability to rapidly metabolize HMOs as the sole carbon source.^61^ Therefore, extrapolating this reasoning to PBNPs, the identification of dietary sources or food waste streams that can offer a diversity of monosaccharide building blocks in polysaccharides and oligosaccharides is highly desirable. Yet, identification of the individual carbohydrate structures comes with numerous analytical challenges. The diversity of monosaccharides brings a complexity of structures, differentiated not only by the composition but also by the position of the many glycosidic linkages connecting the various building blocks. Liquid chromatography coupled with mass spectrometry (LC-MS) has emerged as the preferred tool to both resolve the many isomeric forms of oligosaccharides and decode their composition and structure. A typical workflow for liquid chromatography–mass spectrometry used to identify the oligosaccharides present in a grape sample and measure their abundances is presented in the (Figure 2).

The process of winemaking releases berry skin oligosaccharides from pectin and hemicellulose, and hence the overall composition may be dependent on varietal, enzyme activation during ripeness, winemaking style, and the application of commercial enzyme treatments to increase color.^62^ Whether similar attributes affect the availability of oligosaccharides from the diet is currently unknown. Recent profiling of Chardonnay marc oligosaccharides by Sinrod and colleagues detected up to 36 distinct naturally occurring oligosaccharides between the marc, its fractions, and an extract of the seed. Eleven oligosaccharides shared between the seedless marc and seeds were abundant in hexose and pentose, potentially generated by cell wall arabinogalactan polysaccharides. Five oligosaccharides were unique to the seed but not the seedless marc, while nine additional oligosaccharides were unique to the seedless marc and not the seed. Further analysis of the monosaccharide building blocks of the oligosaccharides indicated that seedless marc oligosaccharides consisted of 11 units that were 81% hexose, predominately (72%) from glucose, with lower amounts of galactose and mannose.^43^ Of interest, the monosaccharide fucose (which is one of the prototypical components of HMOs) was detected at a level of 3.65% followed by arabinose, glucuronic acid, galacturonic acid, ribose, and rhamnose. Although regularly studied separately, fiber/oligosaccharides, polyphenols, and phenolics can associate with one another, and potentially act synergistically, through phenolic/polyphenol inhibition of carbohydrate digestion by brush border enzymes or through the select promotion of beneficial bacterial species.^56,63,64^ Indeed, brush border glucosidases are needed to deglycosylate flavonols prior to absorption.^65,66^

In summary, a comprehensive characterization of PBNP ingredients, such as Chardonnay marc PBNPs, is vital to determine the impact of this dietary component on human health. A major limitation in the interpretation of data from observational studies and dietary intervention trials is the lack of characterization of a food’s bioactives using validated methods that precisely characterize the test products. Given the imprecise nature of measuring food intake, and limited characterization of specific dietary components in food databases, ultimately, the identification of suites of biomarkers will aid in the identification of specific dietary components and patterns that are associated with positive health outcomes.^67,68^

## Potential Contribution of Chardonnay Marc within a Healthy Dietary Pattern

Since the late 19th century, a vast array of PBNPs have been studied for their effects on health, including those recognized as essential macro- and micronutrients. Earlier work in nutritional science defined the essentiality of micronutrients for normal reproduction and development, as well as reducing the risk of specific diseases observed after single-nutrient deficiencies. Albert Svent-Györgyi and others discovered synergistic relationships between essential nutrients, such as vitamin C, and certain flavonoids to include the flavanone from citrus, hesperidin, and the flavanol catechin.^69,70^ By centuries end, observational data from the “monitoring trends and determinants in cardiovascular disease” (MONICA) project and the Seven Countries Study suggested that wine and higher intakes of flavonoid rich foods reduced the risk of development of cardiovascular diseases.^36,71,72^ Research since has supported this concept that certain PBNPs, such as fiber and certain flavonoids, that do not fit the classical definition of “essentiality” are critical for optimal health. The PREDIMED (PREvención con DIeta MEDiterránea) trial provides considerable data on the impact of specific dietary components within a healthy dietary pattern on clinical outcomes and overall cardiometabolic health. For this trial, adults 55–80 years of age with cardiometabolic risk factors were assigned to a reduced fat dietary pattern or a Mediterranean diet with nuts or extra virgin olive oil for 4.8 years. In secondary analyses, those with the highest intake of flavanols (263 mg/d) and flavonols (124 mg/d), respectively, had a 64% and 42% reduction in major cardiovascular events (myocardial infarction, stroke, and cardiovascular death). At study entry, red wine, apples, and peaches contributed most to flavanol intake, while spinach, beans, and onions were the greatest providers of flavonol intake.^73^ Moreover, a reduced risk for the development of type II diabetes was observed for those with the highest levels of flavanol, proanthocyanidin, and hydroxybenzoic acid intake, with protection greatest for obese men and overweight and obese women, with women having higher Mediterranean diet scores.^74^

Renaud and De Longeril paradoxically observed that individuals in countries with higher saturated fat intakes, such as France, also had lower rates of coronary heart disease. Observing that alcohol intake was also inversely associated with reduced platelet aggregation, they postulated that the high intake of wine in these regions protected against coronary heart disease,^36^ a plausible hypothesis as endothelial cell and platelet activation are central in the development of chronic inflammation and cardiovascular diseases.^75^ Using an animal model of coronary cyclic flow reduction, which measures the effects of aspirin on platelet activation induced coronary damage and stenosis, Folts and colleagues demonstrated that apart from alcohol, grape-derived PBNPs could themselves reduce platelet reactivity.^76^ Moreover, the intake of 450 mL/d of Concord purple grape juice by healthy individuals for 1 week reduced the platelet aggregation response to low dose collagen (1 mg/mL) by 77%, while a similar amount of orange or grapefruit juice had no effect on platelet reactivity.^77^ Endothelial activation or endothelial/vascular dysfunction is an early indicator of cardiovascular disease development. Noninvasive measures of vascular function and health, such as flow mediated dilation (FMD), have been associated with cardiovascular risk factors.^78^ Two weeks of daily intake of purple grape juice (approximately 640 mL) improved FMD two hours after intake in coronary heart disease patients.^79^ While intriguing, the grape juice for these experiments was not well characterized, and the relationship between the intake of grape juice derived bioactives and biological effects could not be confirmed.

Since then, numerous short-term randomized controlled dietary intervention trials using well-defined cocoa products have demonstrated that the intake of flavanols improves surrogate outcomes of cardiometabolic health.^80^ Cocoa flavanols include the monomers epicatechin and catechin, procyanidins with up to ten DP of epicatechin units, and the methylxanthine theobromine. A meta-analysis of 15 trials estimated that FMD response improved by 1.17% with cocoa flavanol intake at an optimal level of 95 mg of epicatechin, 20 mg of catechin, and 710 mg of total flavanols.^80^ Additional data demonstrates that purified epicatechin increases vascular function^81−83^ to a greater extent than catechin.^33^ Procyanidin intake apart from the monomeric flavanols does not improve vascular response but can reduce total cholesterol levels and contributes to circulating levels of microbial derived 5-(3′,4′-dihydroxyphenyl)-γ-valerolactone metabolites (γVLM) by 54%.^40^ Taken together, this body of work demonstrates that short-term intake of cocoa flavanols positively modulates physiologically relevant cardiovascular surrogate outcome measures. Ultimately, to demonstrate the impact of any dietary bioactive on health, longer term (years in length) dietary intervention trials that track clinical outcome measures (e.g., myocardial infarction, cardiovascular death, stroke, etc.) are necessary. The recently completed COSMOS trial randomized older adults (mean age 72 years) to either 500 mg of encapsulated cocoa flavanols (containing 80 mg of epicatechin) or placebo for a median of 3.6 years of follow up. The primary end point, a composite of total cardiovascular outcomes (i.e., myocardial infarction, stroke, coronary revascularization, cardiovascular mortality, carotid and peripheral artery surgery, and unstable angina requiring hospitalization) was not statistically significant (*p* = 0.11). However, per protocol, secondary analyses demonstrated a 27% reduction in cardiovascular death, and for those at the highest level of compliance (missed supplementation <8 days/month), cocoa flavanol intake reduced the primary outcome of total cardiovascular events by 15%. Although not a prespecified secondary analysis, major cardiovascular events (myocardial infarction, stroke, and cardiovascular death) was reduced by 16%.^4^ It is noteworthy that this rigorous measure was also the primary outcome for the PREDIMED trial.^4,84^

Although interpreted with caution, the results of the COSMOS trial strongly suggest that cocoa flavanol intake can provide a health benefit. As with the PREDIMED trial, ancillary studies aim to identify potential mechanisms of action and specific populations that may warrant cocoa flavanol supplementation. Certainly, an analysis of the potential impact of diet on the outcomes would be of value, as interactive effects between cocoa flavanols and other dietary components have been reported. This includes methylxanthines within cocoa products, such as theobromine and caffeine,^83,85^ which can enhance both the vascular response and circulating levels of epicatechin metabolites after cocoa flavanol intake.^83^ The synergistic effects from methylxanthines may not be limited to cocoa flavanols, but tea as well, since plasma caffeine levels were associated with FMD response after green tea intake.^86^ It is noteworthy that theobromine has a longer half-life, and along with the caffeine metabolite paraxanthine, inhibition of phosphodiesterase and adenosine receptors are their commonly ascribed bioactivity.^87,88^ Inhibition of phosphodiesterase potentiates the activity of the endothelial derived vasodilator nitric oxide.^89^ Moreover, methylxanthine metabolism by cytochrome P450 (CYP) oxidases such as CYP2E1 can serve as another potential point of dietary interaction as this isoform is inducible by ethanol and inhibited by the intake of the flavonol quercetin.^87,90^ Healthy dietary patterns include increased intake of green leafy vegetables that are high in dietary nitrate. The intake of nitrate (3 mg/kg bw) and cocoa flavanols (2.7 mg/kg bw) significantly enhanced vascular response. Importantly, the vascular response observed was not further enhanced with a higher intake level of nitrate and cocoa flavanols (8.5 mg/kg bw and 10.9 mg/kg bw, respectively).^91^ Taken together, one can postulate that synergies between flavanol intake and other dietary components may explain the inverse associations observed between the intake of foods containing flavanols, such as grape products, tea, apples, and berries, and cardiovascular outcomes in both the PREDIMED and the European Prospective into Cancer (EPIC) trials.^67,73^ The EPIC trial established γVLM as a potential biomarker of flavanol intake, demonstrating a negative correlation between this biomarker and blood pressure. Compared to those with lower γVLM levels, men and women in the highest decile of urinary γVLM had a −1.9 mmHg (95% CI: −2.7; −1.1) and −2.5 mmHg (95% CI: −3.3; −1.8) lower blood pressure, respectively.^67^ Based on current food composition data, the authors estimated that those in the highest levels of flavanol intake consumed at the minimum 148 mg/day of total flavanols and 4 mg/day of epicatechin, with maximal intake estimated at 618 mg/day and 138 mg/day of flavanol and epicatechin, respectively. Mean intakes for both flavanols and epicatechin were estimated at 260 and 36 mg/day, respectively.^67^ While encapsulated cocoa flavanol intake can certainly achieve these levels of intake, a combination of foods within a healthy diet such as apples, berries, wine, and tea may also provide similar flavanol levels.^67,73^ For PBNP ingredients such as Chardonnay marc, with estimated epicatechin levels at approximately 0.9 mg/g, and up to 9 mg/g in the seeds,^43^ a daily serving of 2 teaspoons can provide up to 20% of the mean predicted flavanol intake level achieved by individuals in the EPIC cohort.^67^

## Data from Dietary Intervention Trials of Grape Berries, Juice, Powders, or Marc

The complex mixture of PBNPs within marc, grape fruit, juice and powder has shown positive effects on cardiovascular outcomes in a number of dietary intervention trials (Table 2). Significant improvements in FMD response of 1 to 6.5% from baseline were observed after 2–4 weeks of daily intake of 240–640 mL of commercial “purple” or Concord grape juice (CGJ) by smokers,^92^ those with hypercholesterolemia,^93^ or those with documented coronary artery disease.^79,94^ In individuals with prehypertension, 8 weeks of daily intake of CGJ providing 965 mg of total phenols (units not defined) and lowered systolic and diastolic nocturnal dip blood pressures by 1.4% and 1.5%, respectively,^95^ which is important as a “non-dipping” nighttime blood pressure pattern is a predictor of increased cardiovascular events and mortality.^96^ However, daily CGJ intake did not affect the 24 h ambulatory and office blood pressure, digital microvascular function, or pulse wave velocity (PWV).^95^ Three to four weeks of daily intake of 36–46 g of a standardized freeze-dried grape powder (FDGP) mixed into water increased FMD response in healthy individuals and those with metabolic syndrome. The FDGP, produced from red, green, blue-black seeded, and seedless California table grapes, provided 208–267 mg of total phenols (gallic acid equivalents, GAE), 1.3–1.7 mg of flavonols, and 378–483 mg or approximately 8–10% of the daily value (DV) of potassium.^97,98^ These improvements in vascular outcomes may be due in part to reductions in inflammation and oxidative stress, which positively modulates vascular mediators, such as nitric oxide or the potent vasoconstrictor endothelin-1. Indeed, a reduction in proinflammatory mediators, interleukin (IL)-1β, nicotinamide adenine dinucleotide phosphate oxidase (NADPH) oxidase, and soluble intercellular adhesion molecule (sICAM)-1, have been reported after daily intake of FDGP,^97,99^ grape juice,^100−102^ and black table grapes.^103^

**Table 2:** Cardiometabolic Outcome Results from Dietary Intervention Trials Using Grape Products^a^

grape product	sample size (M/F)	target population	mean age	RCT design	duration	intervention	control	total polyphenols (GAE)	major flavonoids (mg)	results	ref
marc	49 (27/22)	adults, MetS	42.6	crossover	6 wk, 4 wk WO	8 g marc in water	no marc			↓fasting insulin and HOMA-IR; ↑QUICKI	(133)
	27 (27/0)	adults, with one MetS component	43.6	controlled, nonrandomized crossover	4 wk, 4 wk WO	7% marc in burger	no marc in burger	121		↓fasting glucose, HOMA-IR, oxLDL,; ↑plasma vitamin C	(175)
	12 (12/0)	healthy adults	26	randomized, controlled crossover	5 h PP, 1 wk WO	red grape pomace drink	IC control	1562	70% A, 23% F, 4% P	↓GP iAUC insulin vs control	(176)
freeze-dried grape powder	20 (4/16)	obese adults	48.6	controlled, double-blind crossover; short-term intake + PP response with intervention and a high fat and carbohydrate meal	4 wk, 2 wk WO; PP 5 h	60 g	IC, MAC matched, POLY–	297	1.4 E/1.4 C	PP: ↓ET-1 (5 h); ↑NRF2	(99)
	25 (25/0)	adults, MetS	51.3	controlled, double-blind crossover	30 days; 3 wk WO	46 g	IC, MAC matched, POLY–	267	0.14 mg Q, 35 A	↑FMD GP vs control; ↓sICAM-1 vs control	(97)
	19 (7/13)	healthy adults	33.5	low polyphenol diet run-in, followed by single arm intervention	4 wks, 3 wk WO	46 g	POLY– diet	163	0.11 E, 0.67 C	↓Total-C, HDL-C, total BA	(104)
	5 (5/0)	young adults	24	controlled, nonrandomized	3 wks	36 g	IC, POLY–			↑FMD GP vs control, with and without high fat meal	(98)
	44 (0/44)	pre- and postmenopausal women	39.7 (pre), 58.5 (post)	randomized, single blind, controlled crossover	4 wks, 3 wk WO	36 g	IC, POLY–	209		↓triglyceride, LDL-C, apo B and E, TNFα, isoprostane	(105)
	33 (11/22)	obese adults	34.7 (F), 37.1 (M)	randomized, double blind, controlled, crossover	3 wks, 2 wk WO	46 g	MAC matched	0.58 E, 0.88 C, 2.26 Q, 26.9 A		↓GP large LDL-C and particles vs control; ↑GP IL-1b, IL-6 from activated PBMC vs control	(177)
juice	22 (18/4)	documented CAD	64	parallel arm, dose response	4 wks	320, 640 mL PGJ				↑FMD from baseline, no significant difference between intake levels	(94)
	16 (8/8)	adults, hypercholesterolemia	51.6	crossover	2 wks, 2 wk QO	500 mL/day PGJ	RW 250 mL			GJ: ↑FMD GP, ↓sICAM-1 vs baseline	(93)
	64 (44/20)	prehypertensive or stage 1 hypertension	43	double blind, controlled, crossover	8 wks, 4 wk WO	490 mL/day CGJ	IC	965 mg		NS PAT, PWV; ↓glucose, ↑systolic and diastolic nocturnal dip BP	(95)
	20 (12/8)	healthy adults	30.6	single arm	2 wks	490 mL/day PGJ	none			↓PMA, ADP, collagen-induced platelet aggregation, superoxide; ↑NO	(178)
	10 (5/5)	healthy adults	42	crossover	1 wk, 1 wk WO	450 mL/day PGJ	orange and grapefruit juice	1000 mg		GJ: ↓collagen-induced platelet aggregation	(77)
	40 (40/0)	stage 1 hypertension	43	parallel arm, double-blind, controlled	8 wks	420 mL/day PGJ	IC	885		↓systolic and diastolic BP	(179)
	15 (12/3)	documented CAD	62.5	single arm	2 wks	640 mL/day CGJ	none			↑FMD and lag time for LDL oxidation	(79)
	53 (24/29)	healthy adults and hemodialysis patients	62.0 (juice group)	parallel arm	14 days	100 mL/day RGJ	none	640	4.13 Q3R, 3.13 M, 0.02 C, 12.4 mg A	↓LDL-C, apoB100, ox LDL; ↑HDL-C, apoA-I, alpha-tocopherol	(102)
	18 (6/12)	healthy adults and those with type II diabetes	56 (type II diabetes patients)	parallel arm	4 wks	150 mL/day MJ, M wine and M dealcohlized wine	none			NS observed for MJ intake	(180)
	32 (16/16)	hemodialysis patients		randomized, parallel arm		100 mL/day RGJ	none			↓LDL-C, apoB100, NADPH oxidase, MCP-1; ↑HDL-C	(101)
	76	healthy adults	22 (CGJ group); 26 (control group)	double blind, controlled, parallel arm	12 wks	240 mL/day CGJ	IC, no treatment	467	153 mg P+F; 96 A	↑CGJ OGTT vs baseline, no significant between group effects	(181)
	26 (10/16)	healthy smokers	26	randomized, double-blind controlled, crossover	2 wks, 4 wks WO	240 mL/day CGJ	IC, color matched grapefruit juice	473		↑FMD CGJ vs control, post smoking FMD vs control; ↓PWV CGJ vs control	(92)
	26 (10/16)	healthy smokers		randomized, double-blind controlled, crossover	2 wks, 4 wks WO	240 mL/day CGJ	IC, color matched grapefruit juice	473		↓CGJ smoking induced ICAM-1 and PAI-I vs control	(100)
	39 (24/15)	hemodialysis patients	62.9	randomized parallel arm	3 times per wk for 6 mo	100 mL	none	589	152 A	↓total cholesterol from baseline in both groups	(106)
	28 (23/5)	healthy adult runners	39.5	parallel arm	28 days	10 (mL/kg)/d GJ (Isabel, Bourdeux, Concord varietals)	1274	0.7 E, 21.1 C, 36.8 A, 3.43 PB1, 5.29 isoquercetin		↓GJ systolic BP, total-C, LDL-C vs baseline; ↑GJ HDL-C vs baseline	(107)
fruit	69 (14/55)	adults, hypercholesterolemia	51.2	randomized, parallel arm	8 wks	500g Condori red or Sharoodi white grapes	5 servings of fruit	RG 0.652 ± 0.23 vs WG 0.598 ± 0.18 mg/g dw		↓total-C, LDL-C RG and WG vs baseline	(108)
	30 (16/14)	healthy adults	31.5	parallel arm	3 wks, with 4 wk follow up	5 g black table grapes/kg bw	none			↓IL-1β, procoagulant activity, no change in lipid or glucose outcomes with grape intake	(103)

a Abbreviations: A, anthocyanidin; apo, apoprotein; ADP, adenosine diphosphate; BA, bile acids; BP, blood pressure; C, catechin; CGJ, Concord grape juice; E, epicatechin; ET-1, endothelin-1; FMD, flow mediated dilation; F, flavanols; GJ, grape juice; GP, grape pomace; HDL-C, high density lipoprotein-cholesterol; HOMA-IR, Homeostatic Model Assessment for Insulin Resistance; iAUC, incremental area under the curve; IC, isocaloric; ICAM, intercellular adhesion molecule; IL, interleukin; LDL-C, low density lipoprotein-cholesterol; MAC, macronutrient; MetS, Metabolic syndrome; MCP, monocyte chemoattractant protein; M, muscadine; MJ, muscadine juice; NADPH, nicotinamide adenine dinucleotide phosphate; NO, nitric oxide; NRF2, nuclear factor E2-related factor 2; NS, not significant; OGTT, oral glucose tolerance test; oxLDL, oxidized LDL; PAI, plasminogen activator inhibitor; PAT, peripheral arterial tonometry; PB1, procyanidin B1; PBMC, peripheral blood mononuclear cell; PGJ, purple grape juice; PMA, phorbol myristate acetate; POLY–, low polyphenolic; PP, postprandial; PWV, pulse wave velocity; Q, quercetin; QUICKI, quantitative insulin sensitivity check index; RG, red grape; RGJ, red grape juice; RW, red wine; TNF, tumor necrosis factor; WG, white grape; wk, week; WO, washout.

Moreover, grape product intake has consistently improved lipid profiles. Reduced levels of total- and/or LDL-cholesterol have been observed with the short-term intake of FDGP,^104,105^ grape juice,^101,102,106,107^ and red^103,108^ and white grape varietals.^103^ A recent meta-analysis by Lupoli and colleagues of 24 published articles with 587 volunteers in randomized, controlled trials (RCTs) reported −7.6 mg/dL, −6.3 mg/dL, and −14.5 mg/dL reductions in total- and LDL-cholesterol and triglycerides, respectively, with grape product intake (i.e., marc, FDGP, GJ, or grape seed extracts). Moreover, after stratifying by grape product, only those individuals consuming marc and FDGP significantly reduced LDL-cholesterol (−6.3 mg/dL; 95%CI: −11.0, −1.6, *p* = 0.01).^109^ It is important to recognize that a majority of the studies to date have provided limited data from strictly white varietals, with a majority of data derived from red grape varietals or mixtures of varietals containing red varietals that provide the flavonoid subclass anthocyanins not present in significant amounts in white varietals. Anthocyanins provide berry color and are thought to contribute to the positive health outcomes after berry intake,^110,111^ with circulating phenolic anthocyanin metabolites of benzoic and cinnamic acids having the strongest associations with vascular response.^110^

Specific to Chardonnay, Corban et al. recently reported improvements in the reactive hyperemia index (RHI; EndoPAT2000), a measure of digital microvascular function, with 4 months of daily intake of either a defatted and milled Chardonnay grape seed flour or a comparative capsule with matched macronutrients, where the fat was matched with organic grape seed oil. The study participants were recruited from a cardiovascular outpatient population and clinic employees and were relatively young (mean age about 38 years) and predominately female, with cardiovascular risk factors controlled with medication and having “endothelial dysfunction” or a low baseline RHI (<2.0) at study entry.^112^ In this population, systolic blood pressure was reduced 3 mmHg from baseline in both groups. Blood cholesterol levels did not significantly change over time, except for an 8% increase and an 11% decrease in triglycerides in Chardonnay grape seed flour versus comparator, respectively. The provision of 4.6 g of Chardonnay grape seeds each day provided 528 mg of total phenols (GAE) and were composed of 57% fiber, 1.3% monounsaturated fatty acids (MUFA), and 4.3% polyunsaturated fatty acids (PUFA). The comparative capsule was designed to be low in total polyphenols (approximately 4.8 mg/day) but still provided appreciable amounts of fiber (26%), MUFA (2.7%), and PUFA (4.2%). The study results suggest that dietary components beyond the phenolic content may contribute to the observed vascular response. In addition to fiber and PUFA, other bioactives provided by the grape seed oil may include vitamin E and phytosterols.^113^

## Supportive Data from Animal Models

Data from a hypercholesterolemic hamster model, a model that compared to other rodent models best reflects human hepatic cholesterol and bile acid metabolism, demonstrated lower total-, LDL-, and VLDL-cholesterol levels with Chardonnay grape seed flour intake.^114−116^ In the first study, ten animals each consumed for 3 weeks Chardonnay, Cabernet Sauvignon, and Syrah seed flours, all sourced from Sonoma County, California. The Chardonnay seed flour was 1.4- and 1.9-fold greater in total flavonoid content, and 7.2- and 7.6-fold greater in epicatechin content than the Cabernet and Syrah seed flours, respectively. Compared to a control hypercholesterolemic diet, 3 weeks of a hypercholesterolemic diet containing 10% Chardonnay seed flour lowered total-, LDL-, and VLDL-cholesterol by 38%, 56%, and 73%, respectively, and lowered the expression of pro-inflammatory genes for tumor necrosis factor (TNF)-α and monocyte chemoattractant protein (MCP)-1 in adipose tissues, whereas the Cabernet Sauvignon or Syrah seed flour diets did not significantly affect circulating cholesterol levels relative to control.^116^ Overall, the cholesterol lowering effects were related to changes in the expression of genes that regulate hepatic fatty acid, cholesterol and bile acid metabolism, specifically a decrease in the intestinal expression of fibroblast growth factor (FGF) 15, which is regulated by Farnesoid X Receptor (FXR) signaling, a negative regulator of cytochrome P450 (CYP) 7A1. In agreement, animals that consumed the Chardonnay seed flour had increased expression of CYP7A1, a positive regulator of bile acid production.^116,117^ These observed beneficial hepatic effects were repeated in a mouse model of nonalcoholic fatty liver disease (NAFLD), where improvements in insulin sensitivity and a positive modulation of genes associated with oxidative stress, inflammation, and immune response were reported.^115^ For a select number of bacteria, the authors observed an overall reduction in the number of fecal bacteria in hamsters fed a high fat diet with 10% Chardonnay or Cabernet Sauvignon marc. Specifically, relative to control, they observed increased levels of *Bacteroides fragilis* and reduced levels *Lactobacillus* spp and *Bifidobacterium* spp, as well as, reduced Firmicutes/Bacteroidetes (F/B) ratio with 3 weeks of Chardonnay marc feeding. The intake of Cabernet Sauvignon decreased Enterobacteriacae. In addition, significant and strong positive associations between *Lactobacillus* spp and LDL- and total-cholesterol were observed. These observations demonstrate the potential for marc products to influence the microbiome. However, they also illustrate the challenge of associating changes in specific bacterial populations with physiologic/metabolic outcomes as *Lactobacillus* species have been reported as either positive or negative toward cardiovascular health.^118,119^

## Grape Marc PBNPs, Microbial Metabolites, and Health

In its role protecting the host from the environment, the gastrointestinal tract is the largest immune organ in the body, containing trillions of bacteria and associated genes. There is considerable interest in defining diet–gut–microbiota interactions that influence health and an individual’s susceptibility to disease.^119,120^ The central nervous system (CNS) regulates physiological homeostasis through environmental peripheral “sensing” afferents located throughout the body, including the gut, that involuntarily regulates the cardiovascular system and visceral organs via the sympathetic and parasympathetic efferent arms of the autonomic nervous system. Moreover, circulating microbial derived metabolites, such as bile acids and SCFAs, produced from fiber fermentation products, and phenolic/polyphenolic metabolites can regulate gut integrity, along with host immunity and metabolism.^117,121,122^ Therefore, crosstalk between dietary components and the microbiome has the potential to impact overall gut, brain, metabolic, and cardiovascular health.^119,120^ Cholesterol 7α-hydroxylase, also known as CYP7A1, synthesizes bile acids from cholesterol. After conjugation with taurine or glycine the resulting bile salts are stored in the gall bladder and released into the gut with food intake to emulsify dietary fats and aid in both fat and fat-soluble vitamin absorption. Approximately 95% of the bile acids are enterohepatically recirculated with the remaining excreted in the feces. In addition, certain gut bacterial species contain bile salt hydrolase (BSH), bile acid inducible, and bile acid dehydratase enzymes that can produce deconjugated bile acids. Bacterial deconjugation produces more hydrophobic bile acids with limited absorption, and higher fecal excretion.^117^ For those that are absorbed, further transformation in the liver and kidney produces secondary/tertiary bile acids that represent 35% of the bile acids in circulation. These bile acids are of interest for their ability to activate nuclear receptors such as FXR to regulate a number of metabolic processes, including glucose and lipid metabolism.^117,123^

Both dietary fiber and polyphenols can sequester conjugated bile acids, which limits bile acid absorption, making them more accessible for bacterial deconjugation in the colon. This may explain, in part, the lipid lowering effects of grape products.^109,117,123^ Healthy adults that were instructed to add FDGP to a low fiber and polyphenol diet for 4 weeks reduced their total and HDL cholesterol levels. In these individuals, the authors reported a significant reduction in total serum bile acids, with reduced levels of the primary bile acid glycochenodeoxycholic acid (GCDCA) and secondary bile acids glycodeoxycholic acid (GDCA) and taurodeoxycholic acid (TDCA).^104^ Moreover, the changes in both GDCA and TDCA were positively associated with the fecal presence of Actinobacteria, a phylum identified as having glycine conjugation capability as well as BSH activity.^124^ While the mechanism of action could not be defined, these data are consistent with in vitro findings of TDCA and GDCA binding by phenolics typically found in grape products such as gallic acid, catechin, and epicatechin.^125^ Bacterial strains with BSH activity also have the capacity to ferment fiber into SCFAs, such as propionate, and can suppress 3-hydroxy-3-methyl-glutaryl-CoA reductase (HMGR) the rate-limiting enzyme for cholesterol synthesis. SCFAs produced from fiber fermentation may also help clear cholesterol through activation of sterol regulatory element-binding protein (SREBP)-2 to increase hepatic LDL-receptor gene expression.^117^ Additional proposed benefits of SCFAs include stimulation of satiety hormones and improved gut barrier function and glucose homeostasis with overall dietary composition influencing SCFA profile. For example, reduced blood pressures were reported within a DASH dietary pattern when carbohydrate was replaced with either unsaturated fat or plant-based protein.^126^ Although all three diets provided a high level of dietary fiber, circulating levels of total SCFAs, acetate, and propionate were greatest with higher intake of plant protein within the DASH dietary pattern. Moreover, acetate was associated with lower fasting glucose and insulin levels, while propionate and butyrate were associated with lower HDL cholesterol, suggesting that cardiometabolic health is dependent on SCFA species.^127^ Importantly, compared to fecal SCFAs, circulating levels of SCFAs are best associated with markers of metabolic health;^128^ however, fecal SCFA levels may provide additional data on overall gut health, particularly as SCFAs such as butyrate are known to regulate intestinal cell proliferation and overall gut barrier function.^122^ Moreover, there is recognition that gut dysbiosis may have a critical role in the origin of a number of diseases, including neurological conditions such as Parkinson’s Disease, the severity of which may be evaluated through the measurement of both plasma and fecal SCFAs.^129,130^

Studies on the relationship between improved glucose homeostasis and SCFAs after grape marc intake are currently limited. Significantly lower blood pressure, fasting blood glucose, and propionic acid levels were reported when individuals were asked to reduce salt intake through the incorporation of 2 g of Tempranillo marc seasoning into diets providing 810 mg/g of complex polysaccharides and 19 mg/g of fiber for 6 weeks.^131^ Branch chain amino acids play a critical role in the regulation of protein synthesis yet are dysregulated in individuals with obesity and diabetes.^132^ Individuals with at least two components of metabolic syndrome (i.e., BMI > 25 kg m^2^; fasting glucose ≥ 100 mg/dL 1; HDL-cholesterol ≤ 50 mg/dL in women and ≤ 40 mg/dL in men; triglycerides ≥ 150 mg/dL; systolic blood pressure ≥ 130 mmHg or diastolic pressure ≥ 85 mmHg) consumed 8 g of Tempranillo marc every day for 6 weeks. The marc was 68% dietary fiber and 29% total phenols. Overall, marc intake improved fasting insulin levels and sensitivity and increased fecal Bacteroides, while reducing the fecal excretion of the branch chain SCFA isovaleric acid. However, when the study population was split into those that had at least a 10% decline in fasting insulin levels (i.e., “responders”) versus those who did not (i.e., “non-responders”) a reduction in Bacteroides abundance was observed, with no significant change in fecal SCFA levels. Moreover, prior to marc intake the “responder” group had significantly lower Firmicutes abundance and tended to have lower levels of butyrate and valeric acid relative to the non-responders. Although of interest, the participants were asked to participate in a low phenol diet 3 days prior to each study visit, therefore it is unknown what impact the study participants’ habitual diet had on the study outcomes.^133,134^

Key to assessing the potential bioactivity of Chardonnay marc PBNPs is an understanding of the bioavailability, metabolism, tissue distribution, and excretion/elimination of (poly)phenols. This includes the potential interactive effects within either the marc or other dietary components that may affect both absorption and bioactivity. Monomeric flavanols are readily absorbed into the enterocyte, whereas glycosylated flavonols (e.g., quercetin glycosides) are hydrolyzed to the parent aglycone prior to absorption. This is achieved by either lactase phlorizin hydrolase located on the brush border membrane or hydrolysis after transportation into the enterocyte by sodium-glucose co-transporter 1 (SGTL-1) by β-glucosidase.^135^ Flavonol aglycones immediately undergo phase I metabolism (e.g., oxidation, reduction, and hydrolysis reactions), while both flavonols and flavanols undergo phase II metabolism at the level of the intestinal epithelium or in the liver to the major circulating forms as sulfate, glucuronide, and methylated conjugates.^136^ Once absorbed, flavonoids may also be effluxed apically back into the intestine or transported in the bile from the liver into the intestine, followed by deconjugation and colonic metabolism into a series of phenolic metabolites.^137−139^ Larger molecular weight condensed tannins, such as procyanidins greater than trimer, do not undergo gastric hydrolysis^40^ but are accessible for metabolism by gut microbiota. Moreover, rhamnose containing flavonols are not absorbed in the small intestine but are hydrolyzed by bacterial rhamnosidases in the colon.^135^ In addition to bile acid sequestration, polyphenols, fiber, and protein interact with one another, within the food matrix and gastrointestinal track, which may affect bioaccessibility.^140^ Indeed, the concept of “missing” dietary polyphenols is that certain polysaccharide/polyphenol interactions are not characterized by standard polyphenol extraction methodologies.^140,141^ Therefore, polyphenols not bound to dietary fiber are immediately available for absorption, while polyphenols associated with fiber can reach the colon for bacterial metabolism.^140^ Likewise, interactions of phenolics and polyphenols with pancreatic amylase and intestinal brush border enzymes may prevent partial hydrolysis of oligosaccharides in the small intestine.^63^ Finally, considerable interindividual differences in microbial epicatechin metabolism have been reported, with the influence of phylotype on epicatechin bioactivity yet to be explored.^137^

## Role of Computational Methods in Accelerating PBNP Research and Innovation

Developing new classes of upcycled co-products rich in PBNPs with positive impacts on health and environmental sustainability is a significant societal grand challenge. However, this important opportunity is severely constrained by the extraordinary complexity of PBNP mixtures in byproducts produced during processing of plant-based raw materials, coupled with the need for rigorous identification of bioactivity relevant for commercial value creation. The resource intensity associated with successfully executing this type of research and development activity is traditionally associated with the pharmaceutical and flavor and fragrance industries, with complementary basic research efforts existing in the university and government sectors. Transformative advances in science and technology associated with analytical chemistry, biology, biochemistry, genomics, and computational methods offer a new approach to accelerating research and innovation in this area, while simultaneously significantly reducing resource requirements. Central to this approach is integration of a data science strategy enabling high quality predictions based on artificial intelligence capabilities. When successfully executed, this strategy can multiply the value of relatively sparse chemical, biological, and physiological data while simultaneously reducing the risk inherent in human subject experiments required for confirmation of efficacy regarding health or sensory attributes of specific PBNPs. The use of computational methods to accelerate innovation in human health and the gut microbiome has received special attention during the past decade, including the potential influence of diet, and advances in this area can have application for PBNPs.^142^

Simmons et al.^143^ offered a glimpse into the potential of this approach for accelerating research regarding PBNPs present in Chardonnay marc. These investigators integrated publicly available natural products chemistry data sets related to wine grape marc from different varieties, including Chardonnay marc specifically, to investigate their differences and potential implications to human health through a network-based meta-analysis. Chemical composition data was aggregated from publicly available literature, and potential health effects were then identified based on this chemical information and associations between disease states. From currently available data from 132 studies, the analysis was able to differentiate five overabundant compounds present in Chardonnay marc versus other red and white varietal grape marcs. This included the flavanols discussed above, catechin, epicatechin, epigallocatechin, gallocatechin, and proanthocyanidin C1 and determined that gallocatechin was unique to Chardonnay marc. Subsequent analysis with these compounds of 934 studies of 358 disease states and 34 disease classes was able to confirm that these compounds were positively associated with cardiovascular disease outcomes.^143^ Although Chardonnay marc is not widely studied at present, the general framework of network-based meta-analysis utilizing natural products composition information provided a holistic view of the knowledge space for wine grape marc and suggested potential areas of focus for future research programs.

## Deciphering the Flavor Chemistry of Chardonnay Marc: An Epicurean Delight

An exciting and promising discovery for the food and beverage industry is that the extraordinary diversity of bioactive PBNPs that are relevant to human health in Chardonnay marc also contribute to a wide range of desirable sensory attributes. Chardonnay marc has recently gained popularity as a flavorful new food ingredient with its velvety texture, mild astringency, slightly sour taste, and subtle floral and fruit-like aroma attributes. Grape marc has been evaluated as an ingredient in a wide variety of foods. Some examples include baked goods, such as wheat bread and breadsticks, pasta, various beverages, and even chocolate.^144−149^

Some popular health foods and dietary supplements such as pomegranate extract, green tea extract, cocoa extract, and others that are in high demand in the natural foods industry but whose flavor is generally not well accepted by consumers for use directly in foods are often sold in the form of capsules or pills that can be swallowed. Often, this failure of use directly as a food ingredient is due to their strong bitterness, astringency, and various other unpleasant off flavors such as strong vegetative or beany flavors. To include these ingredients directly in foods like beverages or drink powders, much effort has gone into the development of technology that mitigates these undesirable flavor issues. Some of these technologies include encapsulation, absorption of the materials onto protein, and the utilization of bitter-masking agents to increase the consumer enjoyment of these products.^8,150−152^ For Chardonnay marc, such a trend appears to be an exception, as Chardonnay marc is a rich source of PBNPs, similar to other plant-based supplements but has a pleasant flavor, low in bitterness and astringency, and appealing fruity and floral raisin-like retronasal aroma attributes. Not only can Chardonnay marc be used directly as an ingredient in food products and accepted by consumers, but in some cases the addition of Chardonnay marc to foods, such as chocolate, appears to enhance the flavor, which consumers seem to prefer.^149^

When a food is consumed, aroma, taste, and chemosensory sensations are perceived simultaneously and not as discrete events. However, due to the very different nature of the molecules, each requiring a different array of purification and analytical methods, sensory-active compounds are often studied separately. In addition, it has long been overlooked that only a minor fraction of the volatiles present in a food are able to interact with odorant receptors present in the human olfactory system. The molecular sensory science approach termed sensomics, can be used to distinguish the aroma active compounds present in a mixture of odorless volatiles.^153^ Sensomics approaches start with careful isolation of the volatiles by means such as SAFE distillation (solvent assisted flavor evaporation) followed by the application of Aroma Extract Dilution Analysis (AEDA).^154^ Odorants identified with high Flavor Dilution (FD) factors are then accurately quantitated by the application of a gold-standard quantitation method such as Stable Isotope Dilution Assay (SIDA). Finally, the use of an aroma stimulation model can account for interactions among odorants and aroma released from the food matrix. Here, reference odorants are mixed into a model food matrix in the “natural” concentrations measured in the food, with the identification and quantitation of the aroma compounds considered successful if this aroma simulation model mimics the overall food aroma when evaluated through human sensory analysis.^154^

**Figure 3:**
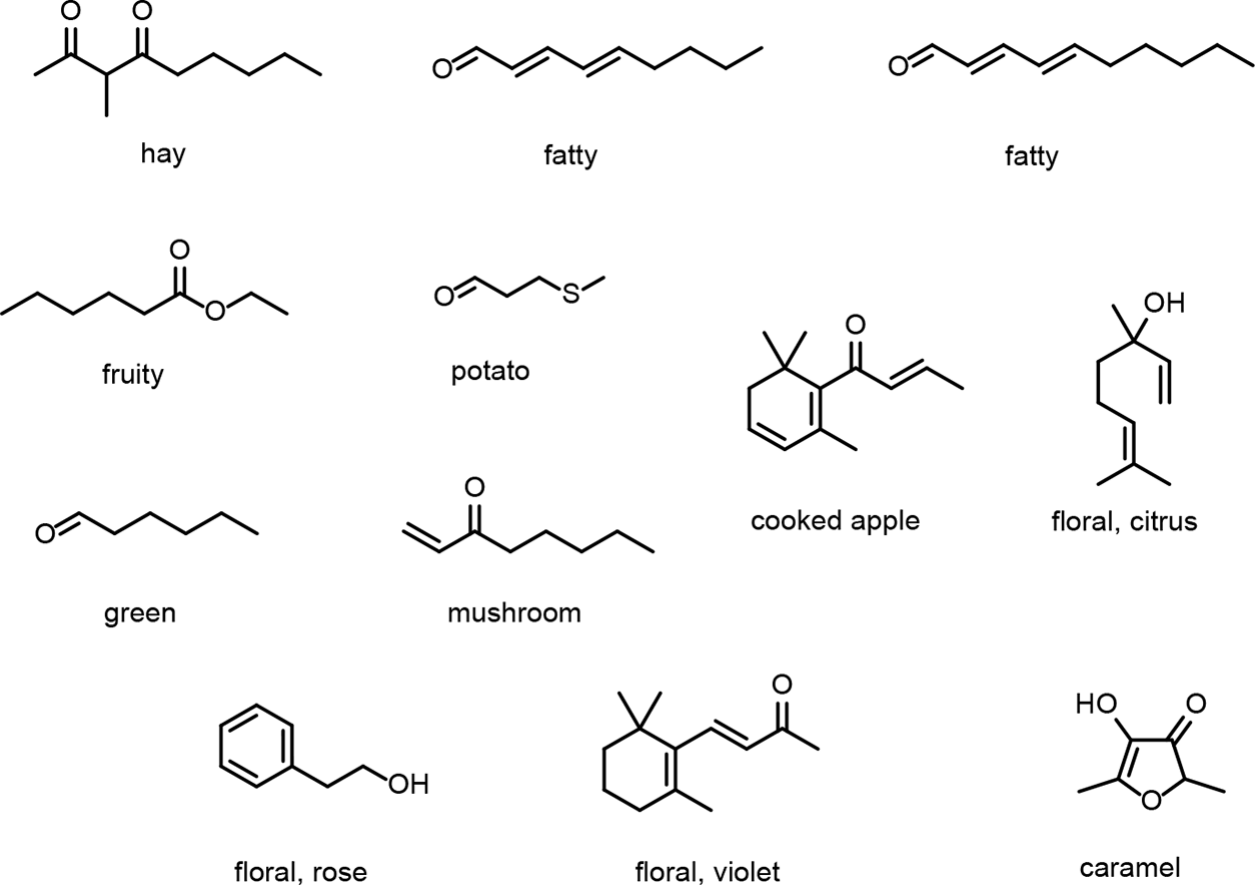
Structures and odor descriptors of odorants identified and quantitated in Chardonnay marc skins with OAVs > 1.^155^

In a recent study applying a sensomics approach to the skin component of Chardonnay marc, thirty-five odorants were identified, with 13 odorants quantitated with SIDA, and odor activity values (OAV) were calculated (Table 3). Odorants with OAVs > 1 included 3-methylnonane-2,4-dione (hay, OAV 5800), β-ionone (floral-violets, OAV 2900), (2*E*,4*E*)-nona-2,4-dienal (fatty, OAV 1200), β-damascenone (cooked apple, OAV 370), hexanal (green, OAV 260), oct-1-en-3-one (mushroom, OAV 200), linalool (floral-citrus, OAV 61), (2*E*,4*E*)-deca-2,4-dienal (fatty, OAV 60), 2-phenylethanol (floral-rose, OAV 16), 3-(methylsulfanyl)propanal (potato, OAV 3.7), 4-hydroxy-2,5-dimethyl-3(2*H*)-furanone (HDMF) (caramel, OAV 2.0), and ethyl octanoate (fruity, OAV 1.1). An odor simulation model prepared using reference odorants with OAVs > 1 was a close match sensorially to the aroma of the Chardonnay marc skins. Accordingly, this collection of odorants was determined to play an important role in the pleasant aroma quality of Chardonnay marc skins.^155^ In another recent study applied to the seed component of Chardonnay marc, forty-three odorants were identified including six with flavor dilution (FD) factors ≥64. The odorant with the highest FD factor was (2*E*,4*E*)-deca-2,4-dienal (fatty) with an FD factor of 1024. Five other odorants were identified with an FD factor of 64 including hexanal (green), linalool (floral, citrus), (2*E*,4*E*)-nona-2,4-dienal (fatty), 3-methylnonane-2,4-dione (hay-like), and 2-phenylethanol (floral, rose).^156^Figure 3 presents the structures and odor descriptors. Based on the results, these odorants may contribute to the overall aroma of the seeds; however, additional quantitative studies are required to support this hypothesis.

**Table 3:** Odorant Name, Odor Quality, and Odor Activity Value (OAV) of Odorants Quantitated in the Skin Component of Chardonnay Marc

odorant name	odor quality	OAV^a^
3-methylnonane-2,4-dione	hay	5800
β-ionone	floral, violet	2900
(2*E*,4*E*)-nona-2,4-dienal	fatty	1200
β-damascenone	cooked apple	370
hexanal	green	260
oct-1-en-3-one	mushroom	200
linalool	floral, citrus	61
(2*E*,4*E*)-deca-2,4-dienal	fatty	60
2-phenylethanol	floral, rose	16
3-(methylsulfonyl)propanal	potato	3.7
HDMF	caramel	2.0
ethyl octanoate	fruity	1.1
pentanoic acid	rancid	<1

a OAV = odorant concentration/odorant threshold in water.^155^

Although a few studies have been conducted on the aroma chemistry of Chardonnay marc, still much of the fundamental aroma chemistry is unknown. This includes potential changes in aroma chemistry that occur during processing such as postharvest handing, drying, milling, and storage. Additional knowledge gaps in the aroma chemistry of Chardonnay marc include agronomic factors such as the potential variation in growth conditions, location, and vintage, highlight areas for future investigations. Even less is known about the taste and chemosensory-active molecules present in Chardonnay marc. Taste and chemosensory-active compounds are generally isolated on the basis of a sensory-directed fractionation focused upon sensory evaluation of fractionated materials. Recently, sensory-directed fractionation of food extracts via chromatographic separations, sensory evaluation, and structural elucidation has led to the discovery of various novel taste and chemosensory-active molecules in foods, including black tea, morel mushrooms, red wine, Swiss cheese, Gouda cheese, cooked crab, and stewed beef.^157−162^ Although the overall process of sensory-guided fractionation (for taste and chemosensory-active molecules) is similar to the process of identifying aroma active compounds in foods, a single methodology does not exist due to the diversity of the chemistry of the molecules. As a result, fractionation schemes are tailored in a case-by-case manner, primarily because these compounds tend to be nonvolatile in nature and structurally diverse. Broadly, a general approach is taken to identify taste and chemosensory-active compounds in foods; however, depending on the chemistry of the molecules, different sequences of separation techniques and analytical methods are employed.

**Figure 4:**
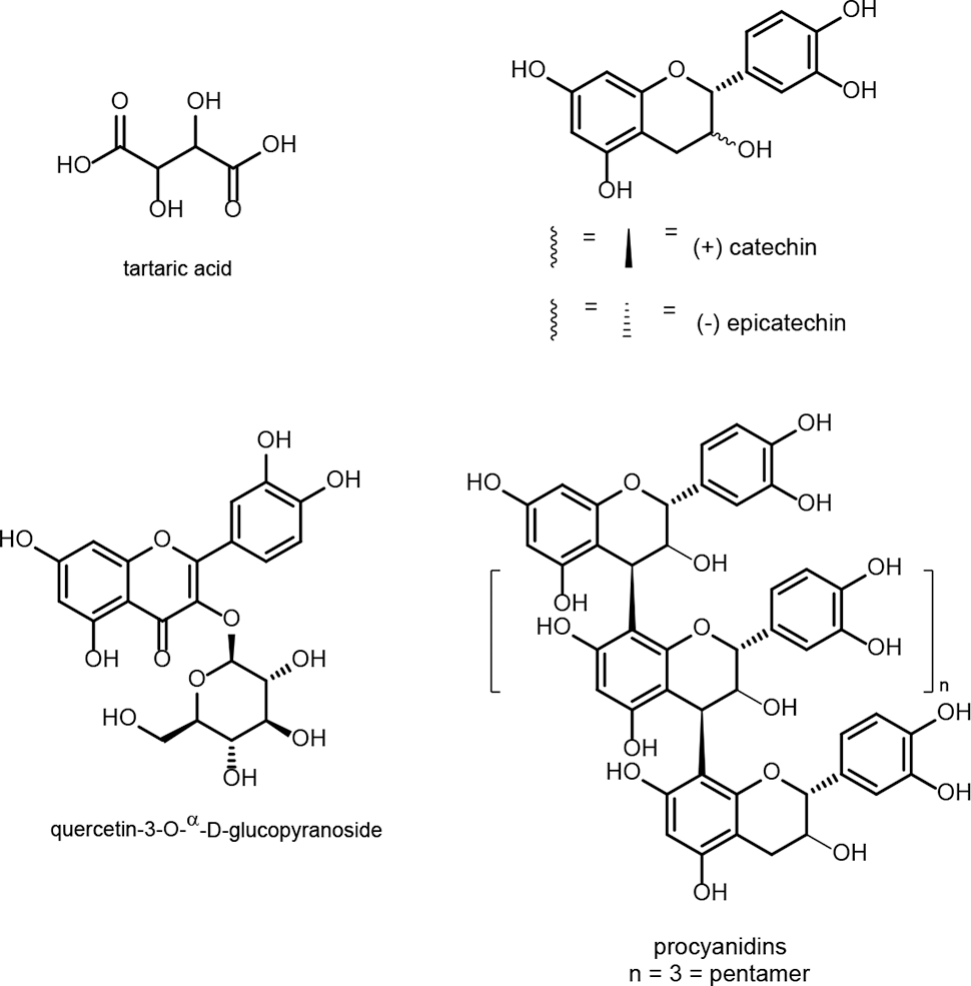
A few selected examples of tastants and chemosensory-active compounds identified in Chardonnay. Tartaric acid is a sour tasting organic acid. The monomeric flavan-3-ols, (+)catechin and (−)epicatechin, and the oligomeric procyanidins are bitter and astringency eliciting compounds. Quercetin-3-*O*-α-d-glucopyranoside elicits a velvety astringent sensory sensation.

So far, there have not been any studies published on the taste or chemosensory-active molecules present in Chardonnay marc. However, some known tastants and chemosensory-active molecules have been reported from grapes, including from Chardonnay. These include the sour tasting organic acids (i.e., succinic, malic, citric, and tartaric acid),^147,163^ the bitter flavan-3-ols and procyanidins,^38,39,43,164−168^ and velvety astringent flavanol glycosides (i.e., quercetin-3-*O*-α-d-glucopyranoside)^38,165,166^ (Figure 4). In addition, amino acids and sugars with known taste activities have been reported.^169,170^ Although, there have been no systematic studies reported on the taste chemistry of Chardonnay marc, based on the unique sensory attributes that it imparts to foods, along with its unique combination of potentially health promoting molecules, Chardonnay marc is a promising source of sensory-active molecules. Additional investigations are clearly warranted to determine the entire suite of sensory-active molecules (odorants, tastants, and chemosensory-active molecules) that contribute to a pleasant eating experience of foods and beverages containing Chardonnay marc. Accordingly, Chardonnay marc is an emerging healthy and flavorful food ingredient that demands further flavor chemistry research.

As dietary recommendations increasingly focus on foods and beverages providing a diverse array of PBNPs, new opportunities for precision health will need to consider both the isolated and interactive effects of these dietary components within any dietary pattern. In parallel, foods and beverages containing PBNPs required to improve public health and enable precision health strategies will need to delight consumers from a sensory perspective. This may include the utilization of upcycled ingredients in foods and beverages that enable a circular bioeconomy, yet can contribute significantly to improving public health and environmental sustainability. For the latter, a scale of market penetration is required in order to drive the use of upcycled ingredients containing PBNPs to volumes sufficient to impact environmental sustainability measures.

Chardonnay marc represents a highly desirable model for development of new upcycled ingredients having PBNP profiles that deliver a combination of health and sensory benefits envisioned to be in high demand from food manufacturers, as they do not require significant additional investments in research and development associated with flavor masking, extraction and fractionation, etc. Recent research focusing on Chardonnay marc has provided additional information regarding the natural products chemistry composition for a wide variety of health and sensory benefits when this marc is included in food and beverage products. Further research is warranted regarding a more detailed understanding of Chardonnay marc and the opportunity it represents as a new model for upcycled ingredients relevant to inclusion in food and beverage products.

To that end, computational data-driven methods have the potential to accelerate research and advance our understanding of how PBNPs affect human health, their mechanism of action, and their optimal processing and integration in our diets. Differential pathway analysis in both preclinical and clinical studies shed light on the differential expression and representation of genes and microbes, hence associating specific genes and taxa to health states and outcomes. Genome-wide models that incorporate metabolic reconstructions, protein–protein interactions, and regulatory networks can provide both mechanistic insights and an in-silico testbed on how dietary changes can modulate microbiota and the availability of metabolic compounds. Machine Learning and Artificial Intelligence can assist in optimizing the production of ingredients that have optimal balance of phenolic content, maximizing the retention and availability of these compounds while processing. Specialized machine learning areas, such as reinforcement learning, can be then applied to create true recommendation systems for the manufacturing of those ingredients and products that can target with higher precision specific health conditions in a synergic and combinatorial manner and hence improve human health in unprecedented ways. Chardonnay marc can be a key resource toward this vision, acting as a model for accelerating innovation converting other byproducts containing PBNPs into upcycled co-products sought after as value added ingredients for use throughout the global food and beverage industry.
